# *N*′-[4-(Dimethyl­amino)benzyl­idene]-3-hydr­oxy-2-naphthohydrazide

**DOI:** 10.1107/S160053680900960X

**Published:** 2009-03-28

**Authors:** Hai-Tao Huang

**Affiliations:** a Pharmacy School, Qiqihar Medical University, Qiqihar 161006, People’s Republic of China

## Abstract

The title compound, C_20_H_19_N_3_O_2_, was obtained by the condensation of 4-(dimethyl­amino)benzaldehyde with 3-hydr­oxy-2-naphthohydrazide. The mol­ecule is approximately planar, with an intra­molecular N—H⋯O hydrogen bond involving the imino H atom and the hydr­oxy O atom. The dihedral angle between the benzene ring and the naphthyl mean plane is 2.72 (13)°. In the crystal structure, symmetry-related mol­ecules are linked by inter­molecular O—H⋯O hydrogen bonds, forming chains propagating in the *c*-axis direction.

## Related literature

For background on compounds obtained by the condensation of aldehydes with benzohydrazides, see: Qiu & Zhao (2008 ▶); Yathirajan *et al.* (2007 ▶); Salhin *et al.* (2007 ▶). For informtaion concerning their biological properties, see: Küçükgüzel *et al.* (2003 ▶); Charkoudian *et al.* (2007 ▶). For similar structures, see: Fun *et al.* (2008 ▶); Liu & Li (2004 ▶); Lei *et al.* (2008 ▶). For bond-length values, see: Allen *et al.* (1987 ▶).
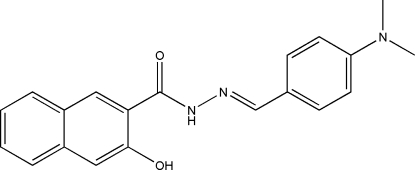


## Experimental

### 

#### Crystal data

C_20_H_19_N_3_O_2_
*M* *_r_* = 333.38
Monoclinic, 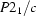

*a* = 8.090 (2) Å
*b* = 15.798 (3) Å
*c* = 13.428 (3) Å
β = 98.978 (3)°
*V* = 1695.2 (7) Å^3^
*Z* = 4
Mo *K*α radiation
μ = 0.09 mm^−1^
*T* = 298 K
0.27 × 0.23 × 0.22 mm

#### Data collection

Bruker APEXII CCD area-detector diffractometer
Absorption correction: multi-scan (*SADABS*; Sheldrick, 1996 ▶) *T*_min_ = 0.977, *T*_max_ = 0.981
13922 measured reflections
3670 independent reflections
1629 reflections with *I* > 2σ(*I*)
*R*_int_ = 0.075

#### Refinement

*R*[*F* ^2^ > 2σ(*F* ^2^)] = 0.068
*wR*(*F* ^2^) = 0.190
*S* = 1.03
3670 reflections
232 parameters
1 restraint
H atoms treated by a mixture of independent and constrained refinement
Δρ_max_ = 0.17 e Å^−3^
Δρ_min_ = −0.17 e Å^−3^

### 

Data collection: *APEX2* (Bruker, 2007 ▶); cell refinement: *SAINT* (Bruker, 2007 ▶); data reduction: *SAINT*; program(s) used to solve structure: *SHELXS97* (Sheldrick, 2008 ▶); program(s) used to refine structure: *SHELXL97* (Sheldrick, 2008 ▶); molecular graphics: *SHELXTL* (Sheldrick, 2008 ▶); software used to prepare material for publication: *SHELXTL*.

## Supplementary Material

Crystal structure: contains datablocks global, I. DOI: 10.1107/S160053680900960X/su2102sup1.cif

Structure factors: contains datablocks I. DOI: 10.1107/S160053680900960X/su2102Isup2.hkl

Additional supplementary materials:  crystallographic information; 3D view; checkCIF report

## Figures and Tables

**Table 1:** Hydrogen-bond geometry (Å, °)

*D*—H⋯*A*	*D*—H	H⋯*A*	*D*⋯*A*	*D*—H⋯*A*
N2—H2*B*⋯O2	0.898 (10)	1.90 (2)	2.644 (3)	139 (2)
O2—H2⋯O1^i^	0.82	1.85	2.651 (3)	165

Symmetry code: (i) 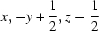.

**Fig. 1.**
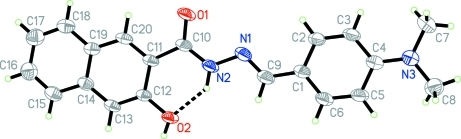
The molecular structure of the title compound, showing the atom-numbering scheme and displacement ellipsoids drawn at the 30% probability level. The intramolecular N2-H2B···O2 hydrogen bond is shown as a dashed line.

**Fig. 2.**
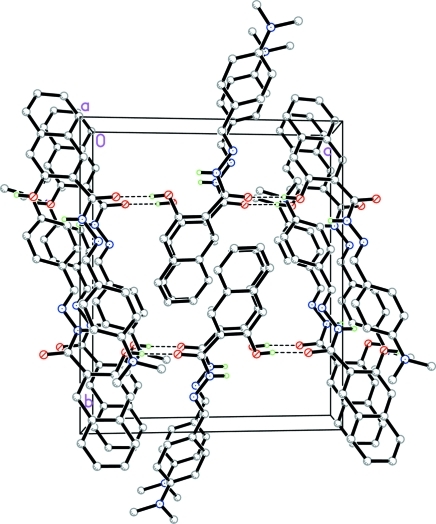
The crystal packing of the title compound viewed along the a axis. The intermolecular O-H···O hydrogen bonds are shown as dashed lines - see Table 1 for details (H-atoms not involved in hydrogen bonding have been omitted for clarity).

## supplementary crystallographic information

### Comment

In recent years compounds derived from the condensation of aldehydes with benzohydrazides have been widely investigated, either for their structures (Qiu & Zhao, 2008; Yathirajan *et al.*, 2007; Salhin *et al.*, 2007) or for their biological properties (Küçükgüzel *et al.*, 2003); Charkoudian *et al.*, 2007). The author reports herein the crystal structure of the title compound, obtained by the condensation of 4-dimethylaminobenzaldehyde with 3-hydroxy-2-naphthohydrazide.

The molecular structure of the title compound is illustrated in Fig. 1. The bond lengths are within normal values (Allen *et al.*, 1987), and are comparable to the values in similar compounds (Fun *et al.*, 2008; Liu & Li, 2004; Lei *et al.*, 2008). The molecule is approximately coplanar, with the dihedral angle between the benzene ring and the naphthyl mean-plane being 2.72 (13)°. There is an intramolecular N-H···O hydrogen bond, involving the imino H-atom and the hydroxyl O-atom (Table 1).

In the crystal structure symmetry related molecules are linked via intermolecular O–H···O hydrogen bonds to form one-dimensional chains propagating in the c direction (Fig. 2 and Table 1).

### Experimental

The title compound was prepared by the condensation of 4-dimethylaminobenzaldehyde (0.1 mol) and 3-hydroxy-2-naphthohydrazide (0.1 mmol) in ethanol (20 ml). The excess ethanol was removed by distillation. The colorless solid obtained was filtered and washed with ethanol. Single crystals, suitable for X-ray diffraction, were obtained on slow evaporation of a solution of the title compound in ethanol.

### Refinement

The imino H-atom was located in a difference Fourier map and refined with the N–H distance restrained to 0.90 (1) Å. The remainder of the H-atoms were positioned geometrically (C–H = 0.93-0.96 Å, O–H = 0.82 Å) and refined using a riding model, with U_iso_(H) = 1.2U_eq_(C) and 1.5U_eq_(O). The ratio of observed to unique reflections is low (44%); this is caused by the fact that the crystal diffracted weakly.

### Crystal data

**Table:**

C_20_H_19_N_3_O_2_	*F*(000) = 704
*M**_r_* = 333.38	*D*_x_ = 1.306 Mg m^−^^3^
Monoclinic, *P*2_1_/*c*	Mo *K*α radiation, λ = 0.71073 Å
Hall symbol: -P 2ybc	Cell parameters from 762 reflections
*a* = 8.090 (2) Å	θ = 2.5–24.3°
*b* = 15.798 (3) Å	µ = 0.09 mm^−^^1^
*c* = 13.428 (3) Å	*T* = 298 K
β = 98.978 (3)°	Block, colorless
*V* = 1695.2 (7) Å^3^	0.27 × 0.23 × 0.22 mm
*Z* = 4	

### Data collection

**Table:**

Bruker APEXII CCD area-detector diffractometer	3670 independent reflections
Radiation source: fine-focus sealed tube	1629 reflections with *I* > 2σ(*I*)
graphite	*R*_int_ = 0.075
ω scans	θ_max_ = 27.0°, θ_min_ = 2.0°
Absorption correction: multi-scan (*SADABS*; Sheldrick, 1996)	*h* = −10→10
*T*_min_ = 0.977, *T*_max_ = 0.981	*k* = −20→20
13922 measured reflections	*l* = −17→17

### Refinement

**Table:**

Refinement on *F*^2^	Primary atom site location: structure-invariant direct methods
Least-squares matrix: full	Secondary atom site location: difference Fourier map
*R*[*F*^2^ > 2σ(*F*^2^)] = 0.068	Hydrogen site location: inferred from neighbouring sites
*wR*(*F*^2^) = 0.190	H atoms treated by a mixture of independent and constrained refinement
*S* = 1.03	*w* = 1/[σ^2^(*F*_o_^2^)]
3670 reflections	(Δ/σ)_max_ = 0.001
232 parameters	Δρ_max_ = 0.17 e Å^−^^3^
1 restraint	Δρ_min_ = −0.17 e Å^−^^3^

### Special details

**Table:**

Geometry. All esds (except the esd in the dihedral angle between two l.s. planes) are estimated using the full covariance matrix. The cell esds are taken into account individually in the estimation of esds in distances, angles and torsion angles; correlations between esds in cell parameters are only used when they are defined by crystal symmetry. An approximate (isotropic) treatment of cell esds is used for estimating esds involving l.s. planes.
Refinement. Refinement of F^2^ against ALL reflections. The weighted R-factor wR and goodness of fit S are based on F^2^, conventional R-factors R are based on F, with F set to zero for negative F^2^. The threshold expression of F^2^ > 2sigma(F^2^) is used only for calculating R-factors(gt) etc. and is not relevant to the choice of reflections for refinement. R-factors based on F^2^ are statistically about twice as large as those based on F, and R- factors based on ALL data will be even larger.

### Fractional atomic coordinates and isotropic or equivalent isotropic displacement parameters (Å^2^)

**Table:**

	*x*	*y*	*z*	*U*_iso_*/*U*_eq_	
O1	−0.0121 (3)	0.25089 (12)	0.13318 (14)	0.0832 (7)	
O2	0.0653 (3)	0.25424 (12)	−0.16764 (12)	0.0674 (6)	
H2	0.0589	0.2496	−0.2289	0.101*	
N1	0.1356 (3)	0.39358 (17)	0.07447 (17)	0.0672 (7)	
N2	0.0872 (3)	0.32477 (18)	0.01253 (17)	0.0675 (7)	
N3	0.4476 (3)	0.76089 (17)	0.2113 (2)	0.0763 (8)	
C1	0.2663 (4)	0.5303 (2)	0.0807 (2)	0.0619 (8)	
C2	0.2427 (4)	0.5531 (2)	0.1772 (2)	0.0701 (9)	
H2A	0.1870	0.5161	0.2144	0.084*	
C3	0.2992 (4)	0.6282 (2)	0.2186 (2)	0.0726 (9)	
H3	0.2785	0.6415	0.2830	0.087*	
C4	0.3873 (4)	0.6862 (2)	0.1683 (2)	0.0615 (8)	
C5	0.4095 (4)	0.6637 (2)	0.0710 (2)	0.0756 (10)	
H5	0.4645	0.7006	0.0333	0.091*	
C6	0.3512 (5)	0.5876 (2)	0.0302 (2)	0.0825 (10)	
H6	0.3699	0.5741	−0.0345	0.099*	
C7	0.4285 (4)	0.7842 (2)	0.3128 (2)	0.0881 (11)	
H7A	0.3154	0.7738	0.3228	0.132*	
H7B	0.4540	0.8432	0.3232	0.132*	
H7C	0.5036	0.7512	0.3599	0.132*	
C8	0.5351 (4)	0.8210 (2)	0.1573 (3)	0.0897 (11)	
H8A	0.6426	0.7985	0.1496	0.134*	
H8B	0.5496	0.8731	0.1944	0.134*	
H8C	0.4711	0.8312	0.0920	0.134*	
C9	0.2072 (4)	0.4523 (2)	0.0329 (2)	0.0716 (9)	
H9	0.2226	0.4443	−0.0336	0.086*	
C10	0.0123 (4)	0.25726 (19)	0.0455 (2)	0.0588 (8)	
C11	−0.0405 (3)	0.18921 (19)	−0.02859 (18)	0.0542 (7)	
C12	−0.0109 (3)	0.18591 (18)	−0.13032 (18)	0.0524 (7)	
C13	−0.0562 (4)	0.11700 (19)	−0.18837 (19)	0.0589 (8)	
H13	−0.0304	0.1152	−0.2535	0.071*	
C14	−0.1407 (3)	0.0483 (2)	−0.1533 (2)	0.0580 (8)	
C15	−0.1911 (4)	−0.0237 (2)	−0.2123 (2)	0.0721 (9)	
H15	−0.1660	−0.0273	−0.2774	0.087*	
C16	−0.2753 (4)	−0.0874 (2)	−0.1759 (3)	0.0830 (10)	
H16	−0.3073	−0.1344	−0.2161	0.100*	
C17	−0.3150 (4)	−0.0836 (2)	−0.0779 (3)	0.0845 (10)	
H17	−0.3747	−0.1274	−0.0540	0.101*	
C18	−0.2665 (4)	−0.0159 (2)	−0.0181 (2)	0.0736 (9)	
H18	−0.2927	−0.0138	0.0469	0.088*	
C19	−0.1767 (3)	0.0512 (2)	−0.0534 (2)	0.0567 (8)	
C20	−0.1228 (4)	0.12143 (19)	0.00589 (19)	0.0598 (8)	
H20	−0.1434	0.1226	0.0721	0.072*	
H2B	0.104 (3)	0.3251 (17)	−0.0520 (10)	0.080*	

### Atomic displacement parameters (Å^2^)

**Table:**

	*U*^11^	*U*^22^	*U*^33^	*U*^12^	*U*^13^	*U*^23^
O1	0.134 (2)	0.0852 (15)	0.0327 (11)	0.0087 (13)	0.0201 (11)	−0.0037 (10)
O2	0.0922 (15)	0.0803 (15)	0.0320 (10)	0.0019 (12)	0.0165 (10)	0.0003 (10)
N1	0.0862 (19)	0.0724 (18)	0.0434 (14)	0.0093 (15)	0.0117 (13)	−0.0071 (13)
N2	0.0881 (19)	0.0812 (19)	0.0343 (13)	0.0019 (16)	0.0136 (13)	−0.0079 (14)
N3	0.090 (2)	0.079 (2)	0.0635 (16)	−0.0053 (16)	0.0226 (15)	−0.0080 (15)
C1	0.074 (2)	0.072 (2)	0.0400 (16)	0.0084 (17)	0.0105 (15)	0.0017 (16)
C2	0.077 (2)	0.087 (2)	0.0491 (18)	−0.0074 (19)	0.0202 (16)	−0.0056 (17)
C3	0.081 (2)	0.096 (3)	0.0452 (17)	−0.007 (2)	0.0232 (16)	−0.0104 (18)
C4	0.0603 (19)	0.076 (2)	0.0488 (17)	0.0130 (18)	0.0099 (15)	0.0022 (17)
C5	0.104 (3)	0.079 (2)	0.0494 (18)	0.003 (2)	0.0292 (17)	0.0065 (17)
C6	0.122 (3)	0.084 (2)	0.0449 (17)	−0.001 (2)	0.0248 (19)	−0.0022 (18)
C7	0.101 (3)	0.097 (3)	0.065 (2)	0.007 (2)	0.0097 (19)	−0.0166 (19)
C8	0.093 (3)	0.087 (3)	0.093 (3)	0.001 (2)	0.024 (2)	0.003 (2)
C9	0.092 (3)	0.082 (2)	0.0416 (17)	0.008 (2)	0.0126 (17)	−0.0021 (18)
C10	0.072 (2)	0.072 (2)	0.0328 (15)	0.0138 (18)	0.0078 (14)	0.0016 (15)
C11	0.0633 (19)	0.0687 (19)	0.0309 (14)	0.0171 (16)	0.0082 (13)	0.0025 (14)
C12	0.0582 (18)	0.065 (2)	0.0344 (14)	0.0108 (16)	0.0090 (13)	0.0047 (14)
C13	0.0668 (19)	0.078 (2)	0.0325 (14)	0.0126 (17)	0.0083 (13)	−0.0040 (15)
C14	0.0569 (19)	0.072 (2)	0.0437 (16)	0.0118 (16)	0.0039 (14)	0.0003 (16)
C15	0.073 (2)	0.091 (3)	0.0513 (18)	0.005 (2)	0.0053 (16)	−0.0084 (18)
C16	0.079 (2)	0.088 (3)	0.078 (2)	−0.010 (2)	0.001 (2)	−0.010 (2)
C17	0.071 (2)	0.097 (3)	0.087 (3)	−0.008 (2)	0.015 (2)	0.002 (2)
C18	0.069 (2)	0.092 (3)	0.063 (2)	0.006 (2)	0.0202 (17)	0.0032 (19)
C19	0.0537 (18)	0.072 (2)	0.0448 (16)	0.0105 (16)	0.0099 (14)	0.0046 (16)
C20	0.069 (2)	0.078 (2)	0.0344 (15)	0.0190 (18)	0.0154 (14)	0.0037 (15)

### Geometric parameters (Å, °)

**Table:**

O1—C10	1.228 (3)	C7—H7C	0.9600
O2—C12	1.376 (3)	C8—H8A	0.9600
O2—H2	0.8200	C8—H8B	0.9600
N1—C9	1.268 (3)	C8—H8C	0.9600
N1—N2	1.387 (3)	C9—H9	0.9300
N2—C10	1.336 (3)	C10—C11	1.481 (4)
N2—H2B	0.898 (10)	C11—C20	1.379 (4)
N3—C4	1.370 (4)	C11—C12	1.424 (3)
N3—C7	1.444 (4)	C12—C13	1.356 (4)
N3—C8	1.444 (4)	C13—C14	1.403 (4)
C1—C6	1.376 (4)	C13—H13	0.9300
C1—C2	1.386 (4)	C14—C15	1.410 (4)
C1—C9	1.436 (4)	C14—C19	1.417 (3)
C2—C3	1.359 (4)	C15—C16	1.349 (4)
C2—H2A	0.9300	C15—H15	0.9300
C3—C4	1.399 (4)	C16—C17	1.404 (5)
C3—H3	0.9300	C16—H16	0.9300
C4—C5	1.392 (4)	C17—C18	1.359 (4)
C5—C6	1.375 (4)	C17—H17	0.9300
C5—H5	0.9300	C18—C19	1.408 (4)
C6—H6	0.9300	C18—H18	0.9300
C7—H7A	0.9600	C19—C20	1.395 (4)
C7—H7B	0.9600	C20—H20	0.9300
			
C12—O2—H2	109.5	H8B—C8—H8C	109.5
C9—N1—N2	114.5 (2)	N1—C9—C1	125.1 (3)
C10—N2—N1	121.8 (2)	N1—C9—H9	117.5
C10—N2—H2B	117.9 (18)	C1—C9—H9	117.5
N1—N2—H2B	120.3 (18)	O1—C10—N2	122.1 (3)
C4—N3—C7	122.3 (3)	O1—C10—C11	120.8 (3)
C4—N3—C8	121.7 (3)	N2—C10—C11	117.1 (2)
C7—N3—C8	116.0 (3)	C20—C11—C12	117.1 (3)
C6—C1—C2	116.3 (3)	C20—C11—C10	116.2 (2)
C6—C1—C9	120.0 (3)	C12—C11—C10	126.6 (3)
C2—C1—C9	123.7 (3)	C13—C12—O2	121.1 (2)
C3—C2—C1	121.5 (3)	C13—C12—C11	120.7 (3)
C3—C2—H2A	119.2	O2—C12—C11	118.2 (3)
C1—C2—H2A	119.2	C12—C13—C14	122.0 (3)
C2—C3—C4	122.6 (3)	C12—C13—H13	119.0
C2—C3—H3	118.7	C14—C13—H13	119.0
C4—C3—H3	118.7	C13—C14—C15	123.3 (3)
N3—C4—C5	121.7 (3)	C13—C14—C19	118.4 (3)
N3—C4—C3	122.4 (3)	C15—C14—C19	118.3 (3)
C5—C4—C3	115.9 (3)	C16—C15—C14	121.1 (3)
C6—C5—C4	120.7 (3)	C16—C15—H15	119.5
C6—C5—H5	119.6	C14—C15—H15	119.5
C4—C5—H5	119.6	C15—C16—C17	120.6 (3)
C5—C6—C1	123.0 (3)	C15—C16—H16	119.7
C5—C6—H6	118.5	C17—C16—H16	119.7
C1—C6—H6	118.5	C18—C17—C16	120.1 (3)
N3—C7—H7A	109.5	C18—C17—H17	119.9
N3—C7—H7B	109.5	C16—C17—H17	119.9
H7A—C7—H7B	109.5	C17—C18—C19	120.7 (3)
N3—C7—H7C	109.5	C17—C18—H18	119.7
H7A—C7—H7C	109.5	C19—C18—H18	119.7
H7B—C7—H7C	109.5	C20—C19—C18	122.6 (3)
N3—C8—H8A	109.5	C20—C19—C14	118.3 (3)
N3—C8—H8B	109.5	C18—C19—C14	119.1 (3)
H8A—C8—H8B	109.5	C11—C20—C19	123.4 (2)
N3—C8—H8C	109.5	C11—C20—H20	118.3
H8A—C8—H8C	109.5	C19—C20—H20	118.3

### Hydrogen-bond geometry (Å, °)

**Table:**

*D*—H···*A*	*D*—H	H···*A*	*D*···*A*	*D*—H···*A*
N2—H2B···O2	0.90 (1)	1.90 (2)	2.644 (3)	139 (2)
O2—H2···O1^i^	0.82	1.85	2.651 (3)	165

Symmetry codes: (i) *x*, −*y*+1/2, *z*−1/2.
